# Worries about memory loss and knowledge on Alzheimer’s disease in community-dwelling elderly from Brazil

**DOI:** 10.1590/S1980-57642011DN05020009

**Published:** 2011

**Authors:** Maria Niures P.S. Matioli, Arnaldo Etzel, João A.G.G. Prats, Wares F. de O. Medeiros, Taiguara R. Monteiro, Alberto de M. Soares

**Affiliations:** 1 Department of Geriatrics of Lusiada University, Santos SP, Brazil;; 2 Department of Clinical Medicine, Santos SP, Brazil;; 3 Medical Sciences Course of Lusiada University, Santos SP, Brazil.

**Keywords:** Alzheimer’s disease, dementia, epidemiological studies

## Abstract

Alzheimer’s disease (AD) is the most common cause of dementia in the elderly. Efforts to determine risk factors for the development of AD are important for risk stratification and early diagnosis. Furthermore, there are no standardized practices for memory screening. Lack of knowledge on AD, perception of memory loss as part of normal aging, and poor socioeconomic conditions may also be implicated in the current situation of dementia.

**Objective:**

To evaluate knowledge of AD in a literate population of elders and correlate these findings with sociodemographic characteristics.

**Methods:**

A descriptive survey design study enrolled 994 volunteers from September 2007 to May 2008 in the city of Santos, São Paulo, Brazil, to answer a brief questionnaire consisting of 8 simple questions about knowledge of AD and worries about memory loss.

**Results:**

Greater knowledge about AD was associated with eight or more years of education, female gender and age between 60 and 70 years. Also, 52.8% of responders (95% CI - 49.5-56.0%) answered that memory loss is part of normal aging and 77.5% (95% CI - 74.7-80.1%) had never sought a doctor to evaluate their memories.

**Conclusion:**

Our study results reinforced that the first line of preventing late diagnosis of dementia is to act in health promotion, especially by targeting subjects older than 70 years of male gender and with lower educational level. It also provided evidence that strategies to promote physician initiative in treating memory problems are also paramount.

## Abstract (pt)

A doença de Alzheimer (DA) é a causa mais comum de demência. Determinar os fatores de risco para o desenvolvimento da DA é importante na estratificação de risco e no diagnóstico precoce. Falta de conhecimento sobre a DA, percepção de perda de memória como parte do envelhecimento normal e más condições de socioeconômicas podem também estar implicadas.

**Objetivo:**

Avaliar o conhecimento sobre DA numa população alfabetizada de idosos e correlacionar esses achados com suas características sociodemográficas.

**Métodos:**

Este estudo descritivo envolveu 994 voluntários de Setembro de 2007 a Maio de 2008 na cidade de Santos, São Paulo, Brasil. Eles se dispuseram a responder um questionário simples composto de 8 questões sobre o conhecimento da DA e preocupações com a perda de memória.

**Resultados:**

Um maior conhecimento sobre DA foi associado à escolaridade >8 anos de estudo, ao gênero feminino e a idade entre 60 e 70 anos. Além disso, 52,8% dos respondedores (IC 95% - 49,5-56,0%) disseram que perda de memória faz parte do envelhecimento normal e 77,5% (IC 95% - 74,7-80,1%) relataram que nunca haviam procurado um médico para avaliação de memória.

**Conclusão:**

Nosso estudo reforça o fato de que a primeira linha de prevenção do diagnóstico tardio de demência é a promoção da saúde, especialmente tendo em foco indivíduos maiores de 70 anos, gênero masculino e maior escolaridade, nossa população de mais alto risco. Também traz evidências de que estratégias para promover a iniciativa dos médicos no cuidado aos problemas de memória são de grande importância.

The elderly population has risen in number globally. World Health Organization (WHO) projections suggest that by 2025, about three-quarters of the 1.2 billion people aged 60 years and older will reside in developing countries.^1^ Current data from developing countries estimate that age-adjusted dementia prevalence in 65 year-olds is ≥5% in certain Asian and Latin American countries, and Alzheimer’s disease (AD) accounts for 60% of this prevalence.^2^

The public health burden of dementia is very high. In the USA 5.4 million suffer from AD, and the direct and indirect costs rise $ 148 billion yearly.^3^ In 2009, it was estimated that there would be 35.6 million people living with dementia worldwide in 2010, increasing to 65.7 million by 2030 and 115.4 million by 2050.^4^ The total estimated worldwide costs of dementia were US$ 604 billion in 2010.^5^

There are no known preventive or curative measures for most types of dementia, including AD.^2^ Early diagnosis of dementia is consistent with the goal of high-quality health care and offers several direct benefits to people with dementia and their caregivers.^6^ Delayed dementia diagnosis however, leads to lost opportunities for treatment and increases patient and caregiver burden.^6^ Knowledge and attitudes about AD contribute to family decision-making about symptoms, diagnosis, treatment, and participation in dementia research.^7^

The present study aimed to evaluate the worries about memory loss and knowledge of AD in a literate elderly population from the Brazilian city of Santos, and to correlate these findings with basic sociodemographic characteristics.

## Methods

### Study design

A descriptive survey design study was performed. A questionnaire was designed including data on sociodemographic characteristics such as age, gender and educational level as predictor variables. Eight “*yes*” or “*no*” questions on knowledge about Alzheimer’s disease were the outcome variables:

[1] Is there someone in your family with AD?
[2] Have you ever heard about AD?
[3] Do you know what AD is?
[4] Do you think that forgetting things frequently is part of normal aging?
[5] Do you have any problem with your memory?
[6] Have you ever sought a doctor to evaluate your memory?
[7] Have you ever taken any medicine for memory?
[8] Do you know if AD is treatable? (Figure). Answers of “yes” to the questions number 3 and/or 8 were considered ‘some knowledge about AD’.

**Figure:**
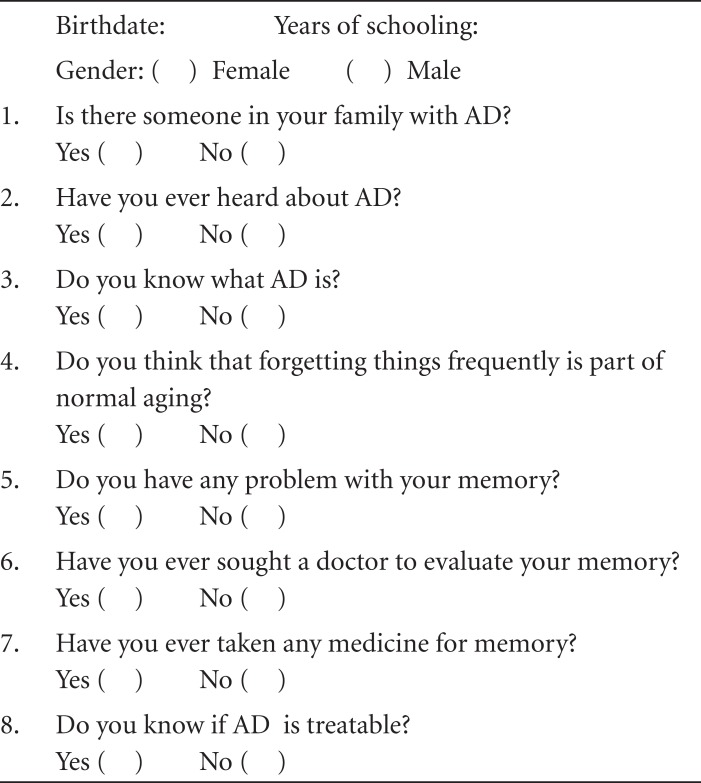
Questionnaire on knowledge about AD (translated to English language).

The study protocol and the written informed consent were approved by the Ethics and Research on Human Beings Committee of the Guilherme Álvaro Hospital.

### Study population

The study subjects were enrolled from September 2007 to May 2008 in the city of Santos SP, Brazil, as a convenience sample. Undergraduate students from the Department of Geriatrics of Centro Universitário Lusíada - UNILUS were assigned to recruit participants in shopping malls, street markets, beaches and Third-age balls and offer them the opportunity to answer the questionnaire. All subjects signed a written informed consent.

### Inclusion criteria

Inclusion criteria were age over 60 years and literacy. Subjects were considered literate if they had the ability to read and fill out the questionnaire by themselves.

### Exclusion criteria

Exclusion criteria were inability to answer, or incomplete filling out of, the questionnaire.

### Statistical analysis

Statistical analysis was performed using Epi info 3.3.2. The results were pooled and analyzed using bivariate analysis of categorical variables, applied using the χ^2^ test and Yates correction as appropriate. Variables with p<0.01, were considered statistically significant according to Bonferroni’s correction. The subjects were classified into two groups for age analysis:

[1] Between 60 and 70 years old;
[2] Greater than 70 years old. Participants were also classified into two groups for schooling analysis:
[1] Between 1 to 8 years of education;
[2] More than 8 years of education.

## Results

Questionnaires were returned by 955 enrolled subjects, of which, 11 questionnaires were excluded because of incomplete filling out. This gave a final total of nine hundred and forty four (944) questionnaires were analyzed. Of the 944 subjects with complete questionnaires, 52% (n=491) were male and 48% (n=453) female with a mean age of 72.2 (±7.2ys) years old. The mean number of years of education was 9.4 (±4.8 ys). The results are shown in Table 1.

**Table 1:** Answer frequency for sociodemographic characteristics.

Questionnaires (n=944)	Male gender (n=491) n (%)	Female gender (n=453) n (%)	p	Between 1 to 8 years of education (n=478) n (%)	More than 8 years of education (n=466) n (%)	p	Age between 60 to 70 years old (n=419) n (%)	Age higher than 70 years old (n=525) n (%)	p
***Most of frequency of "yes" answers***									
Question 2Have you ever heard about AD?	456 (92.9)	441 (97.4)	<0.01	442 (92.5)	455 (97.6)	<0.01	401 (95.7)	496 (94.5)	0.47
Question 3Do you know what AD is?	329 (67.0)	327 (72.2)	0.09	276 (57.7)	380 (81.5)	<0.01	303 (72.3)	353 (67.2)	0.10
Question 4Do you think that forgetting things frequently is part of normal aging?	255 (51.9)	243 (53.6)	0.64	254 (53.1)	244 (52.4)	0.86	209 (49.9)	289 (55)	0.12
Question 8Do you know if AD is treatable?	323 (65.8)	331 (73.1)	0.01	301 (63)	353 (75.8)	<0.01	323 (77.1)	331 (63)	<0.01
***Most of frequency of "no" answers***									
Question 1Is there someone in your family with AD?	429 (87.4)	372 (82.1)	0.03	413 (86.4)	388 (83.3)	0.20	360 (85.9)	441 (84)	0.40
Question 5Do you have any problem with your memory?	348 (70.9)	265 (58.5)	<0.01	299 (62.6)	314 (67.4)	0.13	288 (68.7)	325 (61.9)	0.03
Question 6Have you ever sought a doctor to evaluate your memory?	396 (80.7)	336 (74.2)	0.02	375 (78.5)	357 (76.6)	0.54	342 (81.6)	390 (74.3)	<0.01
Question 7Have you ever taken any medicine for memory?	43259 (88)	387 (85.4)	0.28	421 (88.1)	398 (85.4)	0.86	367 (87.6)	452 (86.1)	0.56

**Question 1. Is there someone in your family with AD?** (For question 1) The majority of subjects (89%; 95% CI - 82.4-87.0%) answered “*no*” to question 1. Gender (p=0.03), years of education (p=0.20) and age (p=0.40) were not statistically associated.

**Question 2. Have you ever heard about AD?** The majority of subjects (95%; 95% CI - 93.4-96.3) answered “*yes*” to question 2. “*Yes*” answers were more frequently associated with female gender (97.4% vs 92.9%; p<0.01) and with more than 8 years of education (97.4% vs 92.5%; p<0.01). Age did not significantly influence frequency of “*yes*” answers (p=0.47).

**Question 3. Do you know what AD is?** The majority of subjects (69.5%; 95% CI - 66.4-72.4) answered “*yes*” to question 3. “*Yes*” answers were more frequently associated with more than 8 years of education (81.5 vs 57.7; p<0.01). Gender (p=0.09) and age (p=0.10) did not influence the answers.

**Question 4. Do you think that forgetting things frequently is part of normal aging?** The majority of subjects (52.8%; 95% CI - 49.5-56.0) answered “*yes*” to question 4. There were no significant differences in the frequency of “yes” answers among gender (p=0.64), years of education (p=0.86) and age (p=0.12).

**Question 5. Do you have any problem with your memory?** The majority of subjects (64.9%; 95% CI - 61.8-68.0%) answered “*no*” to question 5. “No” answers were more associated with male gender (70.9% vs 58.5%; p<0.01). Number of years of education (p=0.13) and age (p=0.03) did not significantly influence the frequency of “*no*” answers (p=0.06).

**Question 6. Have you ever sought a doctor to evaluate your memory?** The majority of subjects (77.5%; 95% CI - 74.7-80.1%) answered “*no*” to question 6. “*No*” answers were more frequently associated with age between 60 and 70 years (81.6% vs 74.3%; p<0.01). The gender (p=0.02) and influence of the number of years of education (p=0.54) did not reach statistical significance.

**Question 7. Have you ever taken any medicine for memory?** The majority of subjects (86.8%; 95% CI - 84.4-88.8%) answered “*no*” to question 7. There was no statistically significant association between gender (p=0.28), years of education (p=0.26) or age group (p=0.56) and frequency of answers.

**Question 8. Do you know if AD is treatable?** The majority of subjects (69.3%; CI 95% - 66.2-72.2) answered “*yes*” to question 8. “*Yes*” answers were more frequently associated with female gender (73.1% vs 65.8%; p=0.01), more than 8 years of education (75.8% vs 63%; p<0.01) and age between 60 and 70 years (77.1% vs 63%; p<0.01).

## Discussion

The present study found that the majority (95%) of the elderly had heard of AD. We also found that only 69.5% of the responders actually knew what AD was, despite the high percentage of “yes” answers to question 1. Women with higher educational level were more likely to have heard about AD, but knowledge on AD was only associated with higher years of education. Stockenrider et al. (1993) also found through questionnaire that 72% of responders had general knowledge of AD.^8^ However, Connell et al. (2009) found that only 42.9% of their subjects had a score over the median in a true-false questionnaire on knowledge about AD.^7^ In line with our findings, Jang et al. (2010) showed that greater knowledge of AD was predicted by higher levels of education and acculturation in a sample of Korean elders.^9^ By contrast, a different group observed that knowledge of AD was very weakly correlated with level of education in a group of Latin American seniors living in Canada.^10^ They also verified no correlation between knowledge level and gender.

The contrasting data on knowledge of AD might be due to the present study’s limitations. Nevertheless, almost every elderly person had heard about AD, but only about 69.5% of them thought they knew what it was. This might reflect the fact that memory loss is poorly addressed in health campaigns, TV, radio and by other communication channels. Disparate findings on the relationship between gender, level of education and knowledge of AD might reflect cultural and educational differences between markedly heterogeneous study populations.

Our data showed 52.8% of subjects believed that memory problems were part of normal aging and other authors had similar findings.^7,11^ Connel et al. (2007) found that 56% of responders correctly stated that Alzheimer’s was not the term for memory loss when we get old.^11^ Later, Connel et al. (2009) showed 54.5% of responders thought “significant loss of memory and mental ability, commonly known as senility, is a normal part of aging” to be a false statement.^7^ Recently, Lee et al. (2010) showed that Korean American immigrants thought memory loss and AD were part of the aging process.^12^ This population seriously lacked knowledge about the treatment, diagnosis and cause of AD, which led many subjects to interpret AD as a form of insanity. A lower knowledge about AD causing stigma was also verified by a different study, which found that feelings of shame associated with family members having AD were more likely to be reported by individuals with lower levels of education, acculturation, and knowledge of AD.^9^ The lack of knowledge about AD may lead to misinterpretation of memory problems and stigmatization of the elderly.

It is troubling that most elders consider that memory loss is part of normal aging, especially given the expected increase in number of people with dementia worldwide over the coming decades, predominantly in low and middle income countries such as Brazil.^5^ Our findings, akin to results of previously published research, reflect a need for health promotion strategies to reinforce in the general population that memory loss is not part of normal aging. This data also supports the vital role that promotion strategies play in preventing stigmatization.

Furthermore, in the present study, 64.9% of subjects reported having no memory problems. Also, the majority (77.5%) reported they had never sought a doctor for memory evaluation. Individuals aged between 60 and 70 years were less likely to report memory problems or seek memory evaluation. The National Memory Screening Day^13^ showed a much larger percentage of women (74%) than men (29%) expressing concerns about their memory, but we failed to confirm this difference between genders in our study.

Congruent with the concept that memory loss is part of normal aging, it is reasonable to expect that the majority of the elderly in our sample would also not seek memory evaluation. These data contrast with data from The “Alzheimer’s Disease: Current Attitudes, Perceptions and Knowledge” survey commissioned by The Alzheimer’s Disease Screening Discussion Group (ADSDG),^14^ which found that more than 90 percent of adults believe screening should occur in the early stages as soon as symptoms are suspected. In addition, about 75% of adults believed it was important to undergo routine screening as part of a physical exam, while 80% of adults aged between 55 and 64 years of age would like to be screened at the next doctor’s appointment.

The population studied has revealed different thoughts on memory problems, probably due to social and cultural aspects. Additionally, our study showed that 69.3% of the responders knew that AD was treatable. Individuals who were women, with higher educational level and aged between 60 and 70 years, were more likely to be aware of this information.

The lack of knowledge about AD, especially its treatment options, deters elderly from undergoing memory evaluation. A recent review described lower level of education, the assumption that cognitive changes were part of normal aging, perception of limited treatment options, and denial of need for/refusal of assessment or forgetting to mention cognitive symptoms to be risk factors, for missed and delayed diagnosis of dementia in primary care.^6^

Our data identified that knowledge held about AD was related to higher level of education as was knowledge of AD’s treatment, where this was also associated with female gender and age between 60 and 70 years. Unfortunately, the majority of responders in our survey thought that forgetting things frequently was part of normal aging, independently of gender, age and educational level. Most men reported having no problems with their memory while most of the elderly between 60 and 70 years old had never sought a doctor to evaluate their memory.

Our study used a questionnaire with simple questions and answers, and the question “knowing what AD is” may not provide reliable information on subject knowledge. Also, illiterates were not included, which could have led to an overestimate of the number of elders knowledgeable about AD, aging, memory screening and for most of the other answers.

In general, our study reinforces that the first line of preventing late diagnosis of dementia is to act in health promotion, especially by targeting subjects older than 70 years of age, of male gender and with lower educational level. The study also provides evidence that strategies to promote physician initiative in treating memory problems are also paramount.
